# Supplementary material for: Personality as a Resource for Labor Market Participation among Individuals with Chronic Health Conditions
Source: Int J Environ Res Public Health. 2020 Aug 27;17(17):6240. doi: 10.3390/ijerph17176240 (PMC7504339; doi:10.3390/ijerph17176240)
Supplement: Supplementary file 1 [file ijerph-17-06240-s001.pdf]

Table S1. Correlations between personality facets of the NEO-PI-R and other baseline characteristics

	1	2	3	4	5	6	7	8	9	10	11	12	13	14
1. Age	1													
2. Sex	0.050***	1												
3. Marital status	-0.159***	-0.027***	1											
4. Educational level	0.181***	-0.027***	-0.026***	1										
5. Anger-hostility	-0.126***	0.007	0.061***	0.116***	1									
6. Self-consciousness	-0.114***	-0.155***	0.084***	0.088***	0.460***	1								
7. Impulsivity	-0.205***	-0.070***	0.086***	0.007	0.379***	0.244***	1							
8. Vulnerability	-0.096**	-0.164***	0.096***	0.120***	0.505***	0.622***	0.313***	1						
9. Excitement-seeking	-0.273***	0.234***	0.075***	-0.060***	0.071***	-0.110***	0.215***	-0.109***	1					
10. Competence	-0.007	0.098***	-0.083***	-0.182***	-0.420***	-0.508***	-0.324***	-0.642***	0.064***	1				
11. Self-discipline	0.122***	0.073***	-0.126***	-0.061***	-0.385***	-0.459***	-0.372***	-0.606***	-0.002	0.598***	1			
12. Deliberation	0.123***	0.052***	-0.098***	-0.082***	-0.360***	-0.236***	-0.503***	-0.342***	-0.180***	0.533***	0.394***	1		
13. Multi-morbidity	0.111***	-0.041***	0.051***	0.086***	0.103***	0.075***	0.050***	0.120***	-0.059***	-0.095***	-0.094***	-0.049***	1	
14. Self-rated health	-0.016	-0.004	0.076***	0.112***	0.177***	0.127***	0.074***	0.189***	-0.045***	-0.167***	-0.156***	-0.077***	0.185***	1

* p-value <0.05; ** p-value <0.01; *** p-value <0.001. Only estimates significant at p<.01 were interpreted.

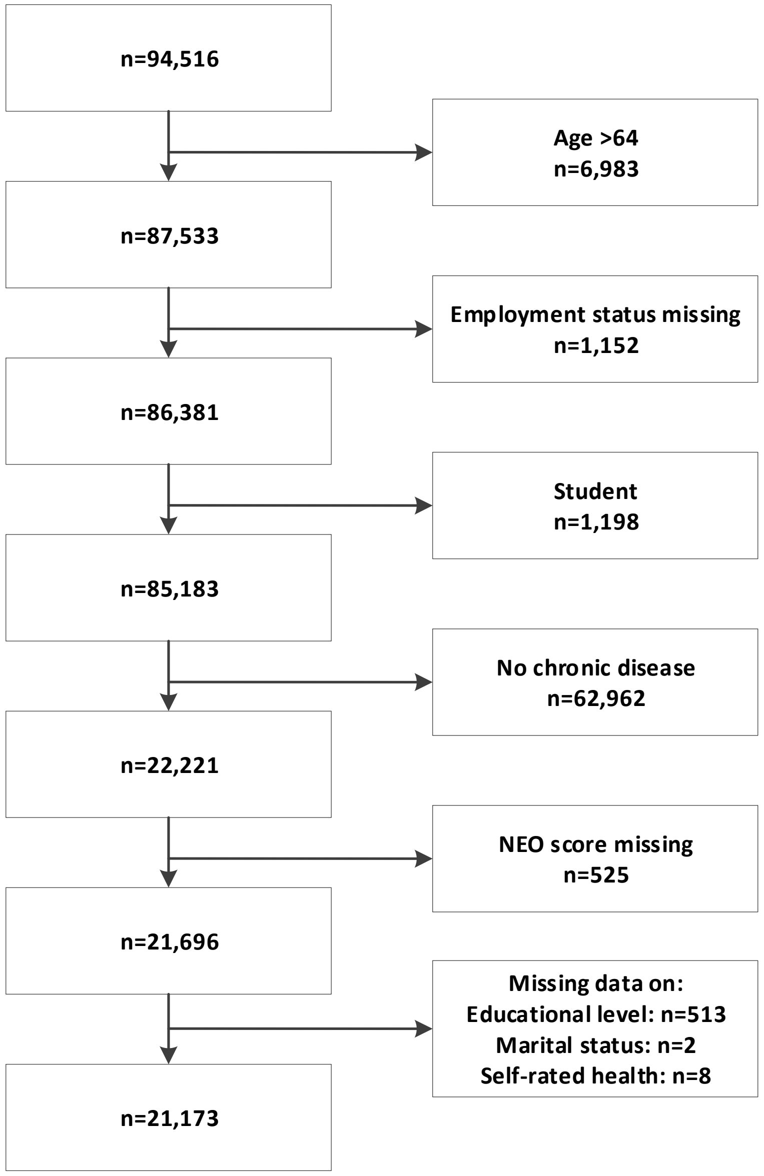

Figure S1. Flow chart of the analytic study sample

Table S2. Associations of personality facets and labour market attachment among individuals with at least one chronic health condition, corrected for socio-demographic factors, multi-morbidity (model 1) and self-rated health (model 2) (n= 21,173)

	**Model 1**	**Model 2**
	OR (95% CI)	OR (95% CI)
Age	0.95 (0.95, 0.96)***	0.95 (0.95, 0.95)***
Gender		
Female	Ref	Ref
Male	2.23 (2.07, 2.41)***	2.30 (2.13, 2.48)***
Marital status		
Living together	Ref	Ref
Not living together	0.71 (0.65, 0.77)***	0.73 (0.67, 0.79)***
Educational level		
High	Ref	Ref
Medium	0.69 (0.62, 0.75)***	0.70 (0.63, 0.76)***
Low	0.37 (0.33, 0.40)***	0.38 (0.35, 0.42)***
Personality facets		
Anger-hostility	0.98 (0.97, 0.99)***	0.98 (0.97, 0.99)***
Self-consciousness	1.01 (1.01, 1.02)**	1.01 (1.00, 1.02)**
Impulsivity	1.03 (1.02, 1.04)***	1.03 (1.02, 1.04)***
Vulnerability	0.98 (0.97, 0.99)***	0.99 (0.97, 1.00)*
Excitement seeking	1.02 (1.01, 1.02)***	1.01 (1.01, 1.02)**
Competence	1.08 (1.07, 1.10)***	1.08 (1.06, 1.09)***
Self-discipline	1.04 (1.03, 1.05)***	1.04 (1.03, 1.05)***
Deliberation	0.96 (0.95, 0.97)***	0.97 (0.96, 0.98)***
Multi-morbidity		
No	Ref	Ref
Yes	0.67 (0.62, 0.73)***	0.74 (0.68, 0.80)***
Self-rated health		
Good		Ref
Poor		0.55 (0.50, 0.59)***

* *p*-value <0.05; ** *p*-value <0.01; *** *p*-value <0.001. Only estimates significant at *p*<.01 or smaller were interpreted.

Table S3. Associations of personality facets and labour market attachment among individuals with at least one chronic health condition stratified by chronic disease and corrected for socio-demographic factors and multi-morbidity; Odds ratio's with their corresponding 95% confidence intervals

	**Cancer** **n=2,931**	**T2DM** **n=1,043**	**Depression** **n=8,299**	**Anxiety disorder** **n=2,658**	**Heart disease** **n=1,246**	**COPD** **n=4,043**	**Musculoskeletal disorder** **n=6,026**
Age	0.92 (0.91, 0.93)***	0.92 (0.91, 0.94)***	0.97 (0.97, 0.98)***	0.98 (0.97, 0.99)***	0.91 (0.89, 0.92)***	0.96 (0.95, 0.97)***	0.92 (0.91, 0.93)***
Gender							
Female	Ref	Ref	Ref	Ref	Ref	Ref	Ref
Male	2.57 (2.04, 3.24)***	2.37 (1.74, 3.22)***	1.86 (1.65, 2.10)***	2.26 (1.81, 2.81)***	2.22 (1.65, 2.98)***	2.58 (2.16, 3.09)***	2.58 (2.24, 2.97)***
Marital status							
Living together	Ref	Ref	Ref	Ref	Ref	Ref	Ref
Not living together	1.01 (0.78, 1.29)	0.70 (0.48, 1.03)	0.64 (0.58, 0.72)***	0.72 (0.59, 0.89)**	0.81 (0.56, 1.16)	0.65 (0.53, 0.79)***	0.71 (0.60, 0.86)***
Educational level							
High	Ref	Ref	Ref	Ref	Ref	Ref	Ref
Medium	0.80 (0.62, 1.03)	0.65 (0.41, 1.03)	0.62 (0.54, 0.71)***	0.65 (0.51, 0.84)**	0.86 (0.58, 1.27)	0.76 (0.60, 0.97)**	0.72 (0.60, 0.86)***
Low	0.34 (0.27, 0.43)***	0.36 (0.24, 0.56)***	0.35 (0.30, 0.40)***	0.35 (0.27, 0.45)***	0.69 (0.48, 0.99)*	0.40 (0.32, 0.50)***	0.39 (0.33, 0.46)***
Personality facets							
Anger-hostility	0.96 (0.94, 0.99)**	0.97 (0.93, 1.01)	0.98 (0.97, 0.99)**	0.97 (0.95, 0.99)*	0.98 (0.94, 1.02)	0.97 (0.95, 0.99)**	0.97 (0.96, 0.99)**
Self-consciousness	1.02 (1.00, 1.05)	1.01 (0.97, 1.06)	1.01 (1.00, 1.03)*	1.01 (0.99, 1.04)	1.02 (0.98, 1.06)	1.03 (1.01, 1.05)*	1.02 (1.00, 1.04)
Impulsivity	1.02 (0.99, 1.06)	1.05 (1.00, 1.10)*	1.03 (1.01, 1.04)***	1.02 (0.99, 1.04)	1.07 (1.02, 1.12)**	1.05 (1.03, 1.08)***	1.04 (1.02, 1.06)***
Vulnerability	1.00 (0.97, 1.03)	0.95 (0.90, 1.00)*	0.99 (0.97, 1.00)	0.97 (0.95, 1.00)	1.01 (0.96, 1.06)	1.00 (0.98, 1.03)	0.98 (0.96, 1.00)
Excitement seeking	1.01 (0.98, 1.03)	1.04 (1.01, 1.08)*	1.02 (1.00, 1.03)**	1.02 (0.99, 1.04)	1.03 (0.99, 1.06)	1.02 (1.00, 1.04)	1.02 (1.00, 1.03)*
Competence	1.09 (1.04, 1.13)***	1.05 (0.99, 1.12)	1.08 (1.06, 1.10)***	1.07 (1.03, 1.11)**	1.09 (1.03, 1.16)**	1.12 (1.08, 1.16)***	1.08 (1.05, 1.11)***
Self-discipline	1.05 (1.02, 1.08)**	1.01 (0.96, 1.06)	1.04 (1.03, 1.05)***	1.04 (1.01, 1.06)**	1.06 (1.01, 1.10)*	1.03 (1.01, 1.06)**	1.05 (1.03, 1.07)***
Deliberation	0.97 (0.94, 1.00)*	0.96 (0.91, 1.01)	0.97 (0.95, 0.98)***	0.97 (0.95, 1.00)*	0.98 (0.94, 1.02)	0.97 (0.95, 1.00)*	0.96 (0.94, 0.98)***
Multi-morbidity							
No	Ref	Ref	Ref	Ref	Ref	Ref	Ref
Yes	0.59 (0.49, 0.71)***	0.49 (0.37, 0.66)***	0.70 (0.63, 0.78)***	0.66 (0.55, 0.80)***	0.64 (0.49, 0.84)**	0.49 (0.42, 0.58)***	0.72 (0.63, 0.82)***

Abbreviations: T2DM; type 2 diabetes mellitus; COPD; chronic obstructive pulmonary disease.

* *p*-value <0.05; ** *p*-value <0.01; *** *p*-value <0.001

Table S4. Associations of personality facets and employment status among individuals with at least one chronic health condition stratified by chronic disease and corrected for socio-demographic factors, multi-morbidity and self-rated health; Odds ratio's with their corresponding 95% confidence intervals

	**Cancer n=2,931**	**T2DM n=1,043**	**Depression n=8,299**	**Anxiety disorder n=2,658**	**Heart disease n=1,246**	**COPD n=4,043**	**Musculoskeletal disorder n=6,026**
Age	0.92 (0.90, 0.93)***	0.92 (0.90, 0.94)***	0.97 (0.97, 0.98)***	0.98 (0.97, 0.99)***	0.90 (0.88, 0.92)***	0.96 (0.95, 0.97)***	0.92 (0.91, 0.92)***
Gender							
Female	Ref	Ref	Ref	Ref	Ref	Ref	Ref
Male	2.62 (2.07, 3.31)***	2.40 (1.76, 3.27)***	1.94 (1.72, 2.19)***	2.45 (1.96, 3.06)***	2.27 (1.68, 3.06)***	2.62 (2.19, 3.14)***	2.66 (2.31, 3.07)***
Marital status							
Living together	Ref	Ref	Ref	Ref	Ref	Ref	Ref
Not living together	1.07 (0.83, 1.37)	0.75 (0.51, 1.10)	0.66 (0.59, 0.74)***	0.75 (0.61, 0.92)**	0.85 (0.59, 1.22)	0.67 (0.55, 0.82)***	0.75 (0.63, 0.89)**
Educational level							
High	Ref	Ref	Ref	Ref	Ref	Ref	Ref
Medium	0.80 (0.62, 1.03)	0.65 (0.41, 1.04)	0.63 (0.55, 0.72)***	0.67 (0.52, 0.86)**	0.91 (0.61, 1.35)	0.78 (0.61, 0.99)*	0.73 (0.61, 0.87)**
Low	0.35 (0.28, 0.45)***	0.36 (0.23, 0.56)***	0.36 (0.31, 0.42)***	0.36 (0.28, 0.47)***	0.76 (0.52, 1.09)	0.42 (0.33, 0.52)***	0.40 (0.34, 0.48)***
Personality facets							
Anger-hostility	0.97 (0.94, 0.99)*	0.98 (0.94, 1.02)	0.99 (0.97, 1.00)	0.97 (0.95, 1.00)*	0.99 (0.95, 1.03)	0.98 (0.96, 1.00)	0.98 (0.96, 1.00)*
Self-consciousness	1.02 (0.99, 1.05)	1.01 (0.97, 1.06)	1.01 (1.00, 1.02)*	1.01 (0.99, 1.03)	1.02 (0.98, 1.06)	1.03 (1.00, 1.05)*	1.02 (1.00, 1.03)
Impulsivity	1.02 (0.99, 1.05)	1.05 (1.01, 1.11)*	1.03 (1.01, 1.04)***	1.02 (0.99, 1.04)	1.07 (1.02, 1.12)**	1.05 (1.03, 1.08)***	1.04 (1.02, 1.06)***
Vulnerability	1.00 (0.97, 1.04)	0.95 (0.90, 1.00)*	0.99 (0.98, 1.01)	0.98 (0.96, 1.01)	1.02 (0.97, 1.06)	1.01 (0.98, 1.04)	0.98 (0.96, 1.00)
Excitement seeking	1.01 (0.98, 1.03)	1.04 (1.00, 1.08)*	1.01 (1.00, 1.03)*	1.01 (0.99, 1.04)	1.03 (0.99, 1.06)	1.01 (0.99, 1.03)	1.02 (1.00, 1.03)*
Competence	1.08 (1.04, 1.13)***	1.05 (0.99, 1.12)	1.08 (1.05, 1.10)***	1.06 (1.02, 1.10)**	1.09 (1.03, 1.16)**	1.11 (1.07, 1.15)***	1.08 (1.05, 1.11)***
Self-discipline	1.05 (1.02, 1.08)**	1.00 (0.96, 1.06)	1.04 (1.02, 1.05)***	1.03 (1.01, 1.06)**	1.06 (1.01, 1.10)*	1.03 (1.01, 1.06)**	1.04 (1.02, 1.06)***
Deliberation	0.97 (0.94, 1.00)	0.96 (0.92, 1.01)	0.97 (0.96, 0.98)***	0.98 (0.95, 1.00)	0.98 (0.94, 1.02)	0.98 (0.95, 1.00)	0.96 (0.94, 0.98)***
Multi-morbidity							
No	Ref	Ref	Ref	Ref	Ref	Ref	Ref
Yes	0.67 (0.55, 0.81)***	0.55 (0.41, 0.75)***	0.77 (0.69, 0.86)***	0.73 (0.60, 0.88)**	0.77 (0.58, 1.03)	0.56 (0.47, 0.66)***	0.78 (0.69, 0.89)***
Self-rated health							
Good	Ref	Ref	Ref	Ref	Ref	Ref	Ref
Poor	0.43 (0.33, 0.55)***	0.54 (0.39, 0.75)***	0.51 (0.46, 0.57)***	0.51 (0.42, 0.63)***	0.45 (0.33, 0.62)***	0.53 (0.44, 0.63)***	0.57 (0.49, 0.66)***

Abbreviations: T2DM; type 2 diabetes mellitus; COPD; chronic obstructive pulmonary disease.

* *p*-value <0.05; ** *p*-value <0.01; *** *p*-value <0.001
